# Evaluating the Relationship between Productivity and Quality in Emergency Departments

**DOI:** 10.1155/2017/9626918

**Published:** 2017-08-03

**Authors:** Hyojung Kang, Nathaniel D. Bastian, John P. Riordan

**Affiliations:** ^1^Department of Systems and Information Engineering, School of Engineering, University of Virginia, Charlottesville, VA, USA; ^2^University of Virginia Health System, Charlottesville, VA, USA; ^3^Department of Supply Chain and Information Systems, Smeal College of Business, Pennsylvania State University, University Park, PA, USA; ^4^Penn State Center for Health Organization Transformation, Pennsylvania State University, University Park, PA, USA; ^5^Emergency Medicine, School of Medicine, University of Virginia, Charlottesville, VA, USA

## Abstract

**Background:**

In the United States, emergency departments (EDs) are constantly pressured to improve operational efficiency and quality in order to gain financial benefits and maintain a positive reputation.

**Objectives:**

The first objective is to evaluate how efficiently EDs transform their input resources into quality outputs. The second objective is to investigate the relationship between the efficiency and quality performance of EDs and the factors affecting this relationship.

**Methods:**

Using two data sources, we develop a data envelopment analysis (DEA) model to evaluate the relative efficiency of EDs. Based on the DEA result, we performed multinomial logistic regression to investigate the relationship between ED efficiency and quality performance.

**Results:**

The DEA results indicated that the main source of inefficiencies was working hours of technicians. The multinomial logistic regression result indicated that the number of electrocardiograms and X-ray procedures conducted in the ED and the length of stay were significantly associated with the trade-offs between relative efficiency and quality. Structural ED characteristics did not influence the relationship between efficiency and quality.

**Conclusions:**

Depending on the structural and operational characteristics of EDs, different factors can affect the relationship between efficiency and quality.

## 1. Introduction

In the United States, emergency departments (EDs) are constantly pressured to improve operational efficiency. As the “safety net,” they provide access to treatment 24 hours per day, seven days per week. Unlike a medical clinic, this emergent care is usually unscheduled and the cost for readiness is high. Importantly, EDs use a substantial amount of these resources for uncompensated care [1]. The Centers for Disease Control and Prevention (CDC) reported that about 20% of all patients, who visited at least one ED, were uninsured [2]. The Centers for Medicare and Medicaid Services (CMS) indicated that nearly 50% all emergency services are uncompensated, and 55% of an emergency physician's time is spent providing care to uninsured or underinsured patients [3]. With high overhead and excessive uncompensated care, EDs must maximize the utilization of their resources.

EDs are also under increasing pressure to improve the quality of care delivered. Over the last two decades, the number of annual ED visits has risen by 44%, while the number of hospital EDs has decreased by 11% [4]. This disequilibrium between supply and demand of healthcare has caused ED overcrowding problems, such as long wait times, patients leaving without being seen (LWBS), and frequent ambulance diversions [5, 6]. Attempting to address quality, insurance companies and healthcare-related government agencies (e.g., CMS) have defined key measures for EDs and have encouraged EDs to collect and report these measures [7]. Using these quality measures, external healthcare organizations provide hospitals with financial incentives or penalties based on ED performance [8, 9]. Some ED quality performance data is also released to the public to address transparency and to help customers evaluate hospitals and providers. In order to gain financial benefits and maintain a positive reputation, EDs need to provide higher quality care than their peer groups.

Simple ratios of inputs to outputs or average costs have primarily been used when analyzing hospital performance [10, 11]. These measures are popular because of their simplicity to calculate and compare to benchmark measures. While these simple measures can provide useful information for individual organizations, they can often give biased, inconsistent information if different organizations are compared and multiple inputs and outputs are considered [12].

Data envelopment analysis (DEA) is a technique that overcomes the limitations of ratio-based analysis for measuring relative efficiency. This technique incorporates multiple inputs and outputs and evaluates performance among a set of peer groups [13]. DEA also permits organizations to identify efficiency frontiers and evaluate the magnitude of divergence from best practice, not from the average shown by regression analysis [14]. Several studies have shown the advantages of using DEA for measuring efficiency over regression analysis [15, 16] and ratio-based analysis [17, 18].

Since Nunamaker's first application of DEA in healthcare [19], the method has been broadly employed for efficiency evaluation of various decision-making units (DMUs) in healthcare. Studies have used DEA to evaluate the efficiency of hospitals [20–26]. It has also been used to compare efficiency performance between academic and nonacademic hospitals and “for profit” and “not for profit” hospitals [14, 27]. DEA has also been adopted to investigate the efficiency of individual physicians and understand factors associated with best practice [28–30].

Some studies have investigated trade-offs between hospital efficiency and quality using DEA. Clement et al. [31] and Valdmanis et al. [32] studied how undesirable outcomes, such as patient mortality and patient safety, negatively affect hospital efficiency. Both studies showed that lower efficiency is associated with poorer quality outcomes. In other words, the study results indicated that in order for less efficient hospitals reduce their undesirable outcomes, they must use their resources more efficiently. Therefore, their relative efficiency improves. Nayar and Ozcan [33, 34] assessed the relationship between hospital efficiency and quality by using quality measures as outputs in DEA models. The findings of these studies indicated that efficiency improvement can be achieved without compromising quality.

The objectives of this study are twofold. The first objective is to assess the relative efficiency of EDs with respect to quality measures. In other words, we aim to evaluate how efficiently EDs transform their input resources into quality outputs. The second objective is to investigate the relationship between the efficiency and quality performance of EDs and the factors affecting this relationship. Oftentimes, it is difficult to achieve both efficiency and quality. Understanding the factors associated with this relationship may help EDs develop improvement strategies and assist external healthcare organizations in determining incentive-based policies.

## 2. Methods

### 2.1. Data

This study combined two data sources to investigate the association between ED efficiency and quality and the factors affecting this relationship. The Emergency Department Benchmarking Alliance (EDBA) data include EDs that share their resource levels and operational measures with their members for benchmarking and performance improvement purposes. The CMS Hospital Compare (HC) database provides a broad range of information on hospital performance for recommended care. From the 2012 CMS HC data, measures associated with ED quality of care were extracted and linked to the 2012 EDBA data.

Of the EDs reporting to the EDBA and CMS, we excluded the ones that had missing data for any of the elements used in our models. We also excluded all pediatric EDs and adult EDs that have overly high (>80,000 visits per year) and low (<20,000 visits per year) patient volume to alleviate the impact of outliers on results.

### 2.2. Data Envelopment Analysis

We performed DEA to evaluate the relative efficiency of EDs, which utilizes a linear programming solution technique. For our study, DMUs are EDs that were included in the combined data. Among various DEA models, this study used the variable returns-to-scale (VRS) model, which was initially proposed by Banker et al. in 1984 [35]. The DEA models may focus on minimizing the use of inputs to produce given outputs (input-oriented) or maximizing the level of outputs using given inputs (output-oriented). This study developed an input-oriented DEA model because we sought to evaluate how efficiently EDs employed their key resources to optimize various ED quality measures. The model's findings are used to determine the relationship between ED efficiency and quality performance.

In DEA, we consider *N* DMU's indexed by *j* = 1, 2,   …  , *N*. Inputs of DMU*_j_* are denoted by {*x*_*ij*_; *i* = 1, 2,   …  , *m*′, *m*′ + 1,   …  , *M*; *j* = 1, 2,   …  , *N*), where *i* = 1, 2,   …  , *m*′ are discretionary input variables and *i* = *m*′ + 1, *m*′ + 2,   …  , *M* are nondiscretionary input variables. Outputs of DMU*_j_* are denoted by {*y*_*rj*_; *r* = 1, 2,   …  , *R*; *j* = 1, 2,   …  , *N*). We solve the following linear programming problem *N* times to obtain the relative efficiency score of each DMU (*θ*_*j*_):

(1) $$\begin{array}{c} minimize\;\theta_{0}-\varepsilon \left(\overset{m^{\prime }}{\underset{i=1}{\sum }}s_{i}^{-}+\overset{R}{\underset{r=1}{\sum }}s_{r}^{+}\right), \end{array}$$

(2) $$\begin{array}{c} subject\;to\;\overset{N}{\underset{j=1}{\sum }}\lambda_{j}X_{ij}+s_{i}^{-}=\theta_{0}X_{{ij}_{0}},\;i=1,2,\;\ldots \;,m^{\prime }, \end{array}$$

(3) $$\begin{array}{c} \overset{N}{\underset{j=1}{\sum }}\lambda_{j}X_{ij}+s_{i}^{-}=X_{{ij}_{0}},\;i=m^{\prime }+1,\;\ldots \;,M, \end{array}$$

(4) $$\begin{array}{c} \overset{N}{\underset{j=1}{\sum }}\lambda_{j}Y_{rj}-s_{r}^{+}=Y_{{rj}_{0}},\;r=1,2,\;\ldots \;,R, \end{array}$$

(5) $$\begin{array}{c} \overset{N}{\underset{j=1}{\sum }}\lambda_{j}=1 \end{array}$$

The objective function (1) seeks the minimum *θ*_0_ that represents the proportional reduction applied to all discretionary inputs of the DMU being evaluated (DMU_0_) to improve efficiency. In (1), the term with a non-Archimedean number (*ε*) is used to address the potential problem with the possible presence of alternate optima and inefficiency by maximizing the slacks (*s* ^+^ and *s* ^−^) without altering the *θ*_0_ value. A DMU*_j_* is efficient if and only if *θ*_*j*_ = 1 and all slacks are zero. Constraints (2)–(4) mean that the linear combinations of the benchmarking group outperforms or is the same as (*θ*_0_ *X*_*j*_0__, *X*_*j*_0__,*Y*_*j*_0__) where constraint (3) is to incorporate nondiscretionary input variable [36].

This study employed four inputs and four outputs to assess the relative efficiency of the EDs, and Table 1 depicts the data elements. Inputs included the number of ED beds including triage areas (BED), physicians' working hours per day (DOC), register nurses' working hours per day (RN), and technicians' working hours per day (TECH). These inputs are the most critical and expensive ED resources that directly impact patient care. When performing DEA, it was assumed that BED is a nondiscretionary variable because it could not be adjusted at the discretion of ED managers, at least in the short-term. Outputs included the following ED quality measures: the rate of left without being seen (LWBS), the percent of heart attack patients given percutaneous coronary intervention within 90 minutes of arrival (PCI), time patients who came to the ED with broken bones had to wait before receiving pain medication (PAIN), and the number of patient visits per day adjusted by patient severity (PPD). LWBS, PCI, and PAIN are key quality metrics that the CMS collects to evaluate the performance of EDs and publishes online to help patients compare hospitals and make informed decisions [37]. PPD was included because it significantly impacts care provider workload, which affects quality of care [38, 39]. It should be noted that the reciprocal of the reported values is used for LWBS and PAIN because lower values of these variables represent better quality of care delivered, while DEA usually assumes that more outputs contribute to higher efficiency.

### 2.3. Multinomial Logistic Regression

To understand whether or not EDs that provide high quality of care also produce the outputs in an efficient manner, we classified the EDs into four categories based on their performance levels. High efficiency refers to an efficient frontier ED (i.e., EDs with an efficiency score equal to one, or *θ* ^∗^ = 1), whereas low efficiency refers to an inefficient ED (i.e., EDs with an efficiency score less than one, or *θ* ^∗^ < 1). High quality refers to an ED whose quality measures (LWBT, PCI, and PAIN) were all better than the national mean (for each metric), whereas low quality refers to an ED with at least one of the three quality measures worse than its national mean. Hence, the EDs are classified into the four categories as follows (depicted in Table 2): high efficiency with high quality (category 1), high efficiency with low quality (category 2), low efficiency with high quality (category 3), and low efficiency with low quality (category 4).

We employed multinomial logistic regression to investigate the relationship between ED efficiency and quality performance and the factors affecting this relationship. EDs in categories 2 and 3 deviated from EDs in category 1, either for quality or for efficiency. However, EDs in category 4 significantly differed from those in category 1. To focus the analysis on identifying ED characteristics that contribute to compromised efficiency or quality, we included only the EDs in the first three categories in the multinomial logistic model. The response variable, ED performance or *Y*, can take on any of *m* = 1, 2, or 3 qualitative values (represented by categories 1, 2, and 3, resp.). Let *π*_*ij*_ denote the probability that the *i*th observation falls in the *j*th category of the response variable; that is, *π*_*ij*_ ≡ Pr(*Y*_*i*_ = *j*), for *j* = 1,   …  , *m*. We have *k* = 6 explanatory variables of interest, *X*_1_, … , *X*_6_, on which the *π*_*ij*_ depend. These six regressors (described in Table 3) represent operational and structural characteristics of the EDs that may be associated with the relationship between ED efficiency and quality performance.

This dependence between ED performance and the six explanatory variables is modeled using a multinomial logistic distribution:

(6) $$\begin{array}{c} \pi_{ij}=\frac{\exp\;\left(\gamma_{0j}+\gamma_{1j}X_{i1}+\cdots +\gamma_{kj}X_{ik}\right)}{1+\sum_{l=1}^{m-1}\exp\;\left(\gamma_{0l}+\gamma_{1l}X_{i1}+\cdots +\gamma_{kl}X_{ik}\right)},\;for\;j=1,\;\ldots \;,m-1, \end{array}$$

(7) $$\begin{array}{c} \pi_{im}=1-\overset{m-1}{\underset{j-1}{\sum }}\pi_{ij},\;for\;category\;m. \end{array}$$

In this multinomial logit model, there is one set of parameters, *γ*_0*j*_, *γ*_1*j*_,   …  , *γ*_*kj*_, for each response category but the baseline. The use of a baseline category (category 1 in our model) is one way to avoid redundant parameters because of the restriction, reflected in (7), that the response category probability for each observations must sum to one. Upon some algebraic manipulation, we get the following model:

(8) $$\begin{array}{c} \ln\frac{\pi_{ij}}{\pi_{im}}=\gamma_{0j}+\gamma_{1j}X_{i1}+\cdots +\gamma_{kj}X_{ik},\;for\;j=1,\;\ldots \;,m-1. \end{array}$$

The regression coefficients in (8) represent effects on the log-odds of membership in category *j* versus the baseline category *m*. These regression coefficients are estimated using the method of maximum likelihood. Note that it is convenient to impose the restriction ∑_*j*=1_^*m*^ *π*_*ij*_ = 1 by setting *γ*_*m*_ = 0 (making category *m* the baseline). This allows us to interpret *γ*_*kj*_ as the effect of *X*_*k*_ on the logit of category *j* relative to category 1 (baseline). In addition, we can form the log-odds of membership in any pair of category *j* and *j*′ (other than category *m*), where the regression coefficients for the logit between any pair of categories are the differences between corresponding coefficients for the two categories. Equation (9) allows us to interpret (*γ*_*kj*_ − *γ*_*kj*′_) as follows: for a unit change in *X_k_*, the logit of category *j* versus category *j*′ is expected to change by (*γ*_*kj*_ − *γ*_*kj*′_) units, holding all other variables constant.

(9) $$\begin{array}{c} \ln\frac{\pi_{ij}}{\pi_{ij^{\prime }}}=\ln\frac{\pi_{ij}/\pi_{im}}{\pi_{ij^{\prime }}/\pi_{im}}=\ln\frac{\pi_{ij}}{\pi_{im}}-\ln\frac{\pi_{ij^{\prime }}}{\pi_{im}}=\left(\gamma_{0j}-\gamma_{0j^{\prime }}\right)+\left(\gamma_{1j}-\gamma_{1j^{\prime }}\right)X_{i1}+\cdots +\left(\gamma_{kj}-\gamma_{kj^{\prime }}\right)X_{ik}. \end{array}$$

## 3. Results

After applying the exclusion criteria, this study used 148 EDs.  Table 4 shows the descriptive statistics of the EDs for some of the data elements. The average number of beds was 32.9. On average, 224.7 work hours for RNs were scheduled per day, while 50.08 and 71.9 work hours were scheduled for physicians and technicians, respectively. The average LWBS rate of the EDs was 2.18% in 2012, while the minimum was 0.2% and the maximum was 14.6%. Of the three procedures (EKG, CT, and XRAY), XRAY was performed most frequently on average.

**Figure 1:**
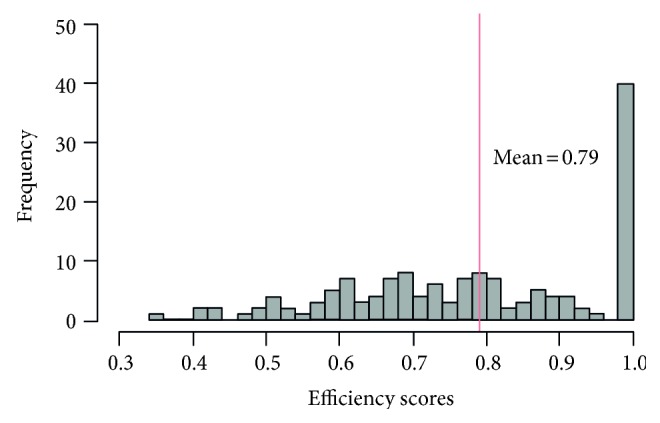
Distribution of DEA scores.

Figure 1 shows the distribution of relative efficiency scores derived from VRS, the input-oriented DEA model for the 148 EDs. The average efficiency score was 0.79, with a standard deviation of 0.173. Of the 148 EDs, 39 EDs (16.9%) achieved an efficiency score of 1 (efficient frontiers), while 12 EDs (8.1%) and 48 EDs (32.4%) obtained relatively high (0.9 ≤ *θ* < 1) and moderate (0.7 ≤ *θ* < 0.9) efficiency scores, respectively. Seven EDs had a lower than 0.5 efficiency score, where the lowest score was 0.34.

For the relatively inefficient EDs, it is important to understand the source of inefficiency.  Table 5 lists their average slacks for inputs (*s*_*i*_^−^^∗^) and amount of excessive inputs (*s*_*i*_^−^^∗^ + (1 *− θ* ^∗^)*x_i_*). The average reduction percentage indicates the average proportion of excessive input to total input. Since BED was used as a nondiscretionary variable in the model, it was not included in the table.

The main source of inefficiencies was TECH, while the contributions of the other two inputs were not significantly different. Among the three inputs, the slack in TECH was observed most frequently in the EDs (80), and the frequencies of slack in DOC (40) and RN (42) were about half of TECH. On average, inefficient EDs had the greatest amount of slack in technicians' working hours (17.3 hours per day), followed by both RNs' (12.3 hours per day) and physicians' working hours (2.64 hours per day). For the inefficient EDs to come up to the efficient frontier, RN needed to be reduced by 86.5 hours, TECH by 41.8 hours, and DOC by 19.4 hours. However, the reduction percentages were similar between DOC (32%) and RN (33%), since the EDs have a larger amount of RN compared to DOC. The percentage was the largest in TECH by 47%.

Table 6 provides a breakdown of the number of EDs that fall into each of the four categories. The distribution of the number of EDs was similar in categories 1, 2, and 3, while about 55% of the entire EDs included in this study were classified to category 4.

The multinomial logistic regression model output is depicted in Table 7. From this regression output, we see that these three statistically significant regressors (EKG, XRAY, and LOS) affect ED performance in terms of the relationship between relative efficiency and quality. For a unit change in EKG, the odds of being in category 2 (high efficiency with low quality) relative to category 1 (high efficiency with high quality) are expected to increase by 19%, while holding all other variables in the model constant. For a unit change in EKG, the odds of being in category 3 (low efficiency with high quality) to category 1 would be expected to increase by 14%, while holding all other variables in the model constant.

On the other hand, a one-unit increase in XRAY is associated with a 17% decrease in the relative risk for being in category 2 versus category 1 and a 16% decrease in the relative risk for being in category 3 versus category 1, when holding all other variables in the model constant.

For a unit change in LOS, the odds of being in category 2 over category 1 would be expected to slightly increase by a factor of 1.05. A unit change in LOS is also associated with a 4% increase in the relative risk for being in category 3 versus category 1.

Contrary to our initial hypothesis, structural ED characteristics, such as teaching status and trauma designation level, did not influence the trade-offs between relative efficiency and quality in EDs.

## 4. Discussion

EDs collect performance measures to identify and manage sources of variation in productivity and quality. Also, EDs benchmark themselves by comparing their performance to peer organizations. A direct comparison of individual outcomes between EDs can result in biased conclusions. A set of simple ratios of input to output can also lead to inconsistent decisions, unless one organization outperforms the other for all measures [40]. DEA helps address these concerns by evaluating the relative performance of an individual organization while simultaneously considering multiple inputs and outputs.

The DEA results indicated that the majority of the EDs in this study had less than optimal production processes. Our results identified the main source of inefficiency as excessive staffing hours. It was estimated that the inefficient EDs needed to reduce technician staffing hours by 47% in order to become efficient. Without fully understanding how these providers function, it is difficult to operationalize this result. For example, this may have occurred because some EDs employed more technicians to replace some clinical and nonclinical roles of nurse staffing. Nursing shortages continue to be a prolonged problem in the United States [41]. Since technicians can assist nurses with a variety of clinical and nonclinical activities while costing EDs less money than nursing staff, the EDs might have hired technicians to fill nursing vacancies in a cost-effective manner. Interestingly, the results also suggest inefficient EDs used 32-33% excessive physician and nurse staffing to create the same level of quality.

For more practical applications of this approach to ED operational management, relative costs of staffing between provider types should be considered. For example, a small reduction in physician hours contributes a greater cost saving because physician hours are significantly more expensive than other staffing hours. Therefore, it would be more cost-effective for inefficient EDs to explore alternatives for excessive physician hours (e.g., increasing middle-level practitioners) prior to managing RN or technician staffing levels. Also, the inclusion of staffing costs in the DEA model may result in different weights between inputs for DMUs, which affects their efficiency score.

The ED classification based on efficiency and quality performance showed that some EDs had trade-offs between efficiency and quality. Among 145 EDs included in this study, 18 EDs utilized their capacity at the optimal level but did not achieve the national average for one of the key quality measures. These EDs could improve the timeliness of emergency care if they had additional resources. On the other hand, 27 EDs outperformed the national average in the three quality measures but did not obtain technical efficiency. These EDs may be under pressure to contain costs in order to remain competitive.

To understand what may influence ED stratification based on efficiency and quality, we performed a multinomial logistic regression analysis that includes both operational and structural factors. We hypothesized that different factors are associated with increasing the odds of losing quality (category 2) or efficiency (category 3). However, the results presented that the same factors affected relative risk by similar magnitudes. In particular, EDs that had a longer LOS were more likely to lose their productivity or fail to deliver timely care for patients with time-sensitive conditions. This may be because LOS tends to be one of the main drivers of hospital costs, which is proportional to the inputs used in determining relative efficiency. Similarly, an increase of LOS may indicate slower care, which can eventually result in reducing quality of care.

The relative frequencies of critical tests in EDs were also associated with ED performance. Interestingly, EDs that performed more EKG procedures per 100 patients were more likely to compromise either productivity or quality. However, an increase in the number of X-ray tests per 100 patients reduced the risk of losing either productivity or quality. The CT test volume showed similar results to X-ray tests, but it was not statistically significant. Further research is needed to understand better why there are differences in the direction of the relationship between EKGs and X-ray/CT tests.

We also hypothesized that teaching status and the level of trauma designation relate to the relationship between relative efficiency and quality. Contrary to our initial hypothesis, the structural characteristics of EDs did not affect sacrificing efficiency or quality. This result implies that EDs can improve their productivity and quality by focusing on reengineering their operational processes to reduce waste in patient flow. Advanced IT systems and standardized protocols may help this improvement without the need to hire additional care providers.

### 4.1. Limitations

This study has several limitations. First, the EDBA and CMS data are self-reporting data, so the data is subject to some inaccuracy that can affect DEA results. However, we believe that our data is trustworthy, since many EDs collect data automatically through an information system (e.g., electronic medical records) and report the data to external organizations for incentive or accreditation purposes.

The inputs used in the DEA could be under- or overestimated. The EDBA data included the scheduled working hours of physicians, RNs, and technicians, and so it might not represent actual worked hours and staffing level. For example, it is possible that care providers were absent, left their jobs, or worked overtime, and these changes were not incorporated into the scheduled hours. However, we believe that the variations do not greatly change the overall care provider working hours or impact our analysis.

The output PPD was adjusted by the CPT to consider the different degrees of resources needed to care for patients with various severity levels. However, the CPT may not be the best proxy to representing the overall severity of the patient population that EDs served. A better patient case-mix adjustment may provide better results.

## 5. Conclusion

Timely care in the ED is crucial for better patient outcomes. Providing timely care is also important for EDs as the payment paradigm changes from fee-for-service payment to value-based payment. Collecting and reporting single measures on timely care can help EDs and external organizations evaluate the performance of EDs and identify areas for improvement. However, using various measures independently can lead to biased conclusions. The DEA approach can help overcome the limitation by providing a relative performance of individual EDs compared to their peer groups and incorporating key inputs.

This study showed that there could be trade-offs between efficiency and quality. Efficient EDs do not always provide high quality of care, and similarly, EDs providing high quality of care do not always achieve technical efficiency. Depending on the structural and operational characteristics of EDs, different factors can affect the relationship. Currently, the payment system associated with the ED performance applies the same criteria to determine incentives or penalties. However, policy makers may need to reflect the differences between the EDs on the payment system so that EDs can be evaluated more fairly and improve their system based on their circumstances.

## Figures and Tables

**Table 1:** Inputs and outputs of DEA models.

	Variables	Definition
Inputs (*x*)	BED	Number of licensed beds contained within the ED including triage rooms
	DOC	Scheduled number of work hours per day of physicians (this does not include work hours of middle-level practitioners and residents)
	RN	Scheduled number of work hours per day of registered nurses
	TECH	Scheduled number of work hours per day of technicians

Outputs (*y*)	LWBS	The ratio of the annual number of patients who leave prior to completion of treatment to the annual number of patients whose visits are registered by the ED (%)
	PCI	Percent of heart attack patients who are given percutaneous coronary intervention within 90 minutes of arrival (%)
	PAIN	Time patients who came to the ED with broken bones had to wait before receiving patient medication
	PPD	Sum of patients who present to the ED for service and are registered by the institution for one calendar year divided by the number of days in the year

**Table 2:** Classification of EDs based on efficiency and quality.

		Quality	
		High	Low
Efficiency	High	Category 1	Category 2
	Low	Category 3	Category 4

**Table 3:** Description of explanatory variables in econometric model.

Variables	Definition
EKG (*X*_1_)	Number of electrocardiograms (per 100 patients) conducted in the ED in 2012.
XRAY (*X*_2_)	Number of X-ray procedures (per 100 patients) conducted in the ED in 2012.
CT (*X*_3_)	Number of computerized axial tomography (CT) scans (per 100 patients) conducted in ED in 2012.
LOS (*X*_4_)	Total ED patient length of stay in 2012.
HOSP (*X*_5_)	Indicator for hospital type in 2012; academic = 1 and nonacademic = 0.
TRAUMA (*X*_6_)	Indicator for whether or not hospital is a Trauma Center in 2012, trauma = 1 or nontrauma = 0.

**Table 4:** Descriptive statistics of the EDBA and CMS data.

Variable	Minimum	Maximum	Mean	Standard deviation
BED	12	75	32.90	12.85
DOC	24	192	50.08	20.59
RN	92	480	224.7	82.89
TECH	8	296	71.90	46.53
PPD	28	172	92.76	31.72
LWBT	0.2	14.6	2.18	1.70
PCI	95	100	96.91	3.94
PAIN	22	264	60.49	22.15
LOS	98	495	183.5	49.43
EKG	10	90	33.27	12.62
XRAY	11	76	55.34	13.92
CT	10	83	26.65	11.69

**Table 5:** Slacks and excessive inputs from DEA model.

	Slacks		
	DOC	RN	TECH
	(*s*_2_^−^^∗^)	(*s*_3_^−^^∗^)	(*s*_4_^−^^∗^)
Frequency (*s*_*i*_^−^^∗^ > 0)	40	42	80
Average amount of slacks	2.64	12.30	17.31

	Excessive inputs		
	DOC	RN	TECH
Average amount	19.4	86.5	41.8
Average reduction percentage	32%	33%	47%

**Table 6:** Breakdown of ED category classifications.

		Quality	
		High	Low
Efficiency	High	21 EDs	18 EDs
	Low	27 EDs	82 EDs

**Table 7:** Multinomial logistic regression model output.

Category/factor	Coefficient	Relative risk ratio	Standard error	*z*	*p*
1	(Baseline category)				
2					
EKG	0.17	1.19	0.07	2.36	0.01^∗^
XRAY	−0.19	0.83	0.06	−2.91	0.00^∗^
CT	−0.03	0.97	0.06	−0.43	0.66
LOS	0.05	1.05	0.02	2.21	0.02^∗^
HOSP	1.30	3.68	1.06	1.23	0.21
TRAUMA	1.59	4.88	1.03	1.54	0.12
(Intercept)	−2	0.14	3.13	−0.64	0.52
3					
EKG	0.13	1.14	0.07	1.94	0.05^∗^
XRAY	−0.17	0.84	0.06	−2.79	<0.01^∗^
CT	−0.05	0.96	0.06	−0.77	0.44
LOS	0.04	1.04	0.02	2.15	0.03^∗^
HOSP	0.77	2.16	0.99	0.78	0.43
TRAUMA	0.66	1.94	0.97	0.69	0.49
(Intercept)	0.05	1.05	2.87	0.02	0.98

^∗^Statistical significance at *α* = 0.05.
